# Effectiveness of Prophylactic Tranexamic Acid in Reducing Blood Loss in Children Undergoing Open Nephrectomy for Wilms Tumor: A Randomized Controlled Study

**DOI:** 10.7759/cureus.85374

**Published:** 2025-06-04

**Authors:** Sateesh Verma, Shraddha Agarwal, Prem R Singh, Neel K Mishra

**Affiliations:** 1 Department of Anaesthesiology and Critical Care, King George's Medical University, Lucknow, IND

**Keywords:** intraoperative blood loss, pediatric, perioperative fluid management, tranexamic acid, wilms tumor

## Abstract

Introduction

Nephrectomy for Wilms tumor is often associated with significant intraoperative blood loss. Tranexamic acid (TXA) is effective in reducing intraoperative bleeding during a wide variety of surgeries in adults and cranial and cardiac surgeries in children. This study aimed to determine the effect of TXA in reducing blood loss in nephrectomy for Wilms tumor, a common malignancy among children. The primary objective was to assess its effect on the amount of intraoperative blood loss. Secondary objectives were to measure packed red blood cells (PRBC) transfusion requirements and evaluate intraoperative hemodynamic parameters.

Materials and methods

In this randomized controlled study, 30 children belonging to the TXA group (intervention arm) received a loading dose of intravenous TXA 50 mg/kg over 10 minutes immediately after induction, followed by its infusion at 5 mg/kg/h. Thirty patients belonging to the normal saline (NS) group (control arm) received intravenous NS of the same volume, without any active drug.

Results

The TXA group has significantly lower intraoperative surgical blood (216.33 vs 306.33 mL; p=0.001). The mean volume of PRBC transfused was also lower in the TXA group (167.0 vs 199.8 mL; p=0.045). The serum lactate at the end of surgery in the TXA group was lower than the control group (1.82 vs 2.63 mmol/L; p=0.001). Mean arterial blood pressure (MAP) and heart rate were similar in both groups. No adverse events were identified either intraoperatively or in the immediate postoperative period.

Conclusion

Intravenous TXA is significantly effective in decreasing intraoperative blood loss in children undergoing nephrectomy.

## Introduction

Wilms tumor, or nephroblastoma, is one of the most common surgical malignancies in children [1-3]. Its diagnosis is usually made in children aged 2-3 years. The standard treatment modality is primary nephrectomy, with chemotherapy administered either before or after surgery. Surgical procedure for Wilms tumor is often associated with a considerable amount of intraoperative blood loss and potentially leads to a large volume of fluid resuscitation and blood transfusions [4,5]. Excessive blood loss may result in hypotension, tachycardia, hypothermia, metabolic acidosis, prolonged surgical time, transfusion reactions, need for postoperative ventilation, coagulopathies, and death [6,7]. Pediatric patients possess a smaller circulating blood volume relative to adults, rendering them more vulnerable to the hemodynamic effects of even modest blood loss [8]. Furthermore, some children with Wilms tumor may already have anemia because of malignancy and chemotherapy [9]. Considering these issues, there is a need for some intervention to reduce intraoperative bleeding in children undergoing nephrectomy for Wilms tumor.

Tranexamic acid (TXA) is a synthetic analog of lysine amino acid. It binds to lysine-binding sites on plasminogen and reduces its transformation into plasmin, which is responsible for degrading fibrin clots [10]. A lot of research has been done for TXA on adult surgical patients, mostly in the areas of heart and orthopedic surgeries, which showed a significant reduction in the amount of surgical blood loss and the need for transfusions [11,12].

The effect of TXA in the pediatric age group was investigated in open heart surgery, scoliosis correction, craniosynostosis repair, and adenotonsillectomy procedures. TXA effectively lowers bleeding and the need for blood transfusions during these surgeries without raising the risk of thromboembolic events [13-16]. Even though clinical research evidence supports the use of TXA in these types of pediatric surgeries, its role in surgical oncologic procedures like Wilms tumor surgery needs confirmation by clinical research.

The aim of this study was to determine the efficacy of prophylactic TXA in reducing blood loss in children undergoing unilateral nephrectomy for Wilms tumor. The primary objective was to quantify intraoperative surgical blood loss. Secondary objectives were to investigate the amount of blood transfusion, intraoperative hemodynamic parameters, serum lactate, urine output, and adverse events in both groups.

## Materials and methods

Study design and patient inclusion criteria

This study was conducted after obtaining approval from our Institutional Ethics Committee with Ref. Code: XXI-PGTSC-IIA/P7. It was also registered with the Clinical Trial Registry of India (No. 2024/09/073921). The study was conducted between 13 September 2024 and 5 May 2025 at our institution, which is a tertiary healthcare center in India.

The study was designed as a prospective, randomized controlled one to study the effect of prophylactic injection of TXA on bleeding in patients undergoing open surgical nephrectomy.

We included children with Wilms tumor aged less than 10 years with or without prior chemotherapy, planned for elective open nephrectomy surgery. Children with coagulopathy, hepatic dysfunction, elevated serum creatinine, and recent nonsteroidal anti-inflammatory drug use were excluded.

Randomization, concealment, and group allocation

An independent statistician generated a random number sequence by using the RAND function in Microsoft Excel (version 2010, Microsoft Corp., Redmond, WA), and it was used to randomize the recruited patients into one of two groups with a 1:1 allocation ratio. The randomization results were kept in sealed envelopes for concealment and were opened at the time of anesthesia induction. Healthcare personnel who opened the sealed envelope and prepared the unlabeled drug infusion was not involved with data collection and data analysis. Both the patient and the attending anesthesiologist responsible for data collection were blinded to the nature of the drug intervention.

According to group allocation, patients had received either intravenous TXA (TXA group) or 0.9% NS (NS group) infusion just after anesthesia induction.

Anesthesia and intraoperative management

Intraoperatively, all patients were monitored with continuous ECG, pulse oximetry, invasive arterial blood pressure, temperature monitoring, urine output, and arterial blood gas (ABG) analysis. For induction, patients were administered fentanyl (2.0 mcg/kg) and propofol (2-3 mg/kg) along with atracurium (0.5 mg/kg). General anesthesia was maintained by sevoflurane (approximately 1 MAC) and fentanyl boluses (1.0 mcg/kg) every hour. Volume-controlled ventilation, along with a positive end-expiratory pressure (PEEP) of 5 cmH_2_O, was used in every patient. A thoracic epidural catheter was placed at T10-T11 level for perioperative analgesia, and it was supplemented with 0.125% bupivacaine every 90 minutes with 0.3 mL/kg volume. A radial artery catheter was placed after induction to facilitate continuous blood pressure monitoring and ABG sampling.

Immediately after induction, patients in the TXA group received a loading dose of intravenous TXA (50 mg/kg) diluted up to 20 mL with NS, administered over 10 minutes, followed by an infusion of 5 mg/kg/h. Patients in the NS group received intravenous NS of the same volume as those in the TXA group, without any active drug. This TXA dose regimen was based on a previous study that found it safe and effective to use [16].

The surgical approach was uniform in all cases. A transverse incision was made, and nephroureterectomy along with paraaortic and paracaval node sampling was performed, followed by en bloc removal of the kidney and tumor. These surgeries were performed by three senior surgeons who had experience of more than 10 years in pediatric surgery.

All patients received Ringer's lactate solution for intraoperative fluid requirements. Its administered volume was calculated by the Holiday Segar formula in components of maintenance rate, fasting deficit, and blood loss replacement. Fluid replacement was also guided by arterial blood pressure, urine output, and serum lactate level. Packed red blood cells (PRBC) transfusion was started if the hemoglobin level reached or fell below 8.0 g/dL in ABG. PRBC transfusion was stopped if the hemoglobin level of 10 g/dl was achieved at the end of surgery. Vasopressor injection norepinephrine had to be started if a 25% reduction in mean arterial blood pressure (MAP) is persisting despite boluses of Ringer's lactate or PRBC.

We applied the following set of formulas for assessment of blood volume lost (BVL) used in previous studies [13-15]:

\[\text{BVL} = \frac{\text{ERCM}_{\text{lost}}}{\text{EBV factor} \times \frac{\text{Hct}_{\text{preop}}}{100}}\]

where EBV factor (estimated blood volume factor) is 80 mL/kg for children less than 12 months and 75 mL/kg for children >12 months. Hct means hematocrit.

ERCM_lost_ was calculated from the formula given below:

\[\text{ERCM}_{\text{lost}} = \text{ERCM}_{\text{preop}} + \text{ERCM}_{\text{transfused}} - \text{ERCM}_{\text{postop}}\]

where ERCM (estimated red cell mass) is calculated by the following:

\[\text{ERCM} = \text{EBV factor} \times \frac{\text{Hct}}{100}\]

\[\text{ERCM}_{\text{transfused}} = \text{PRBC (ml transfused)} \times \frac{\text{Hct}_{\text{transfused PRBC}}}{100}\]

Data recorded for this study were age, weight, gender, American Society of Anesthesiologists (ASA) status, tumor size, hemoglobin, platelet count, international normalized ratio (INR), serum creatinine, and duration of surgery. Intraoperative total blood loss (mL), total PRBC volume transfused, urine output, lactic acid levels by ABG analysis (every 30 minutes), heart rate, and MAP were also recorded. Patients were monitored 48 hours postoperatively for any adverse events like deep vein thrombosis, seizures, and altered mental status.

Sample size calculation and statistical analysis

The sample size calculation was based on a previous study by Goobie et al. [16], where the mean blood loss was 65±26 mL/kg in the TXA group and 119±67 mL/kg in the control group, yielding a mean difference of 54 mL/kg. The sample size was calculated by assuming a 0.05 level of significance (Zα/2=1.96) and 80% power (Z [1 β]=0.84). The calculation yielded a required sample size of 28.75 patients per group, which was rounded up to 30 patients in each group.

All collected data were analyzed with the help of SPSS software (version 20.0; IBM Inc., Armonk, NY). The findings were expressed as mean (±SD) or percentages. Statistical tests used during data analysis were the chi-square test for categorical data, the Mann-Whitney U test for non-parametric data, and the independent student’s t-test for numerical data. If the obtained p-value was <0.05, then the difference was considered statistically significant.

## Results

**Figure 1:**
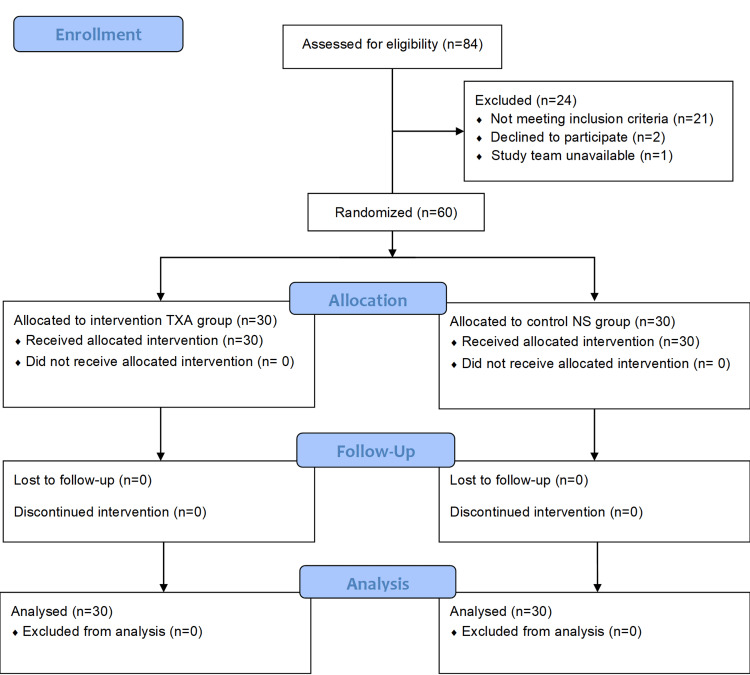
Consolidated Standards of Reporting Trials flow diagram TXA: tranexamic acid; NS: normal saline.

A total of 84 children for elective nephrectomy were assessed for eligibility, out of which 60 were randomized into two groups, with 30 patients in each, as shown in Figure 1.

The mean and ratio values of different demographic parameters like age, weight, height, gender distribution, BMI, and ASA physical status were comparable in both groups. Additionally, there was no statistically significant difference between the preoperative laboratory results, including blood hemoglobin, platelet count, INR value, serum creatinine, and tumor size among the two groups, as shown in Table 1.

**Table 1:** Patient data and baseline characteristics of the study population ^a^Data are expressed as mean±standard deviation. ^b^Data are expressed as ratios. ^c^Chi-square test was used. In the rest of the data, student's t-test was used. TXA: tranexamic acid; NS: normal saline; BMI: body mass index; ASA grading: American Society of Anesthesiologists; INR: international normalized ratio; g/dL: gram per deciliter; mcl: microliter.

Variables	TXA group (n=30)	NS group (n=30)	p-Value	Chi-square/t-value
Age (years)^a^	3.20±1.86	2.53±1.52	0.133	1.52
Weight (kg)^a^	11.01±3.17	10.58±3.11	0.598	0.53
Height (cm)^a^	80.87±13.27	79.33±18.79	0.716	0.37
Male/Female^b^	12/18	14/16	0.75	0.07^c^
BMI^a^	16.88±3.11	17.45±3.89	0.534	-0.63
ASA grade II/III^b^	27/3	21/9	0.106	2.60^c^
Tumor size (mm)^a^	68.63±23.18	68.67±20.73	0.995	0.01
Preoperative hemoglobin (g/dL)^a^	11.34±1.27	12.10±1.36	0.29	-2.24
Preoperative platelet count (10^5^/mcL)^a^	2.65±0.65	2.92±0.56	0.87	-1.74
Preoperative INR^a^	1.19±0.12	1.22±0.11	0.261	-1.14
Serum creatinine^a^	0.72±0.16	0.60±0.14	0.063	2.93

Total intraoperative blood loss was 216.33±37.74 mL in the TXA group and 306.43±24.98 mL in the NS group, and the difference was statistically significant (p=0.001). Total PRBC volume transfused was lower in the TXA group (167.01±24.97 mL vs 199.78±23.32 mL, p=0.045). The TXA group showed significantly lower serum lactate levels at the end of surgery, and the difference among groups was statistically significant. Total duration of surgery, intraoperative urine output, and blood hemoglobin at the end of surgery were comparable between the groups. No incidence of adverse event or need for vasopressor infusion was found in any group, as shown in Table 2.

**Table 2:** Intraoperative blood loss, blood transfusion, and other parameters ^a^Data are expressed as mean±SD, and student's t-test was used. *Significant p-value. TXA: tranexamic acid; NS: normal saline.

Variables	TXA group (n=30)	NS group (n=30)	p-Value	t-Value
Duration of surgery(minutes)^a^	132.03±19.37	138.04±13.75	0.138	-1.72
Total intraoperative blood loss (mL)^a^	216.33±37.74	306.43±24.98	0.001*	-10.89
Intraoperative transfused PRBC volume (mL)^a^	167.01±24.97	199.78±23.32	0.045*	2.05
Postoperative Hb (g/dL)^a^	10.94±2.13	9.8±2.22	0.057	2.03
Serum lactate by ABG (mmol/L)^a^	1.82±0.27	2.63±0.31	0.001*	-10.69
Urine output (mL)^a^	49.02±26.18	39.50±22.14	0.135	1.52
Vasopressor support	0	0	-	-
Adverse event	0	0	-	-

**Figure 2:**
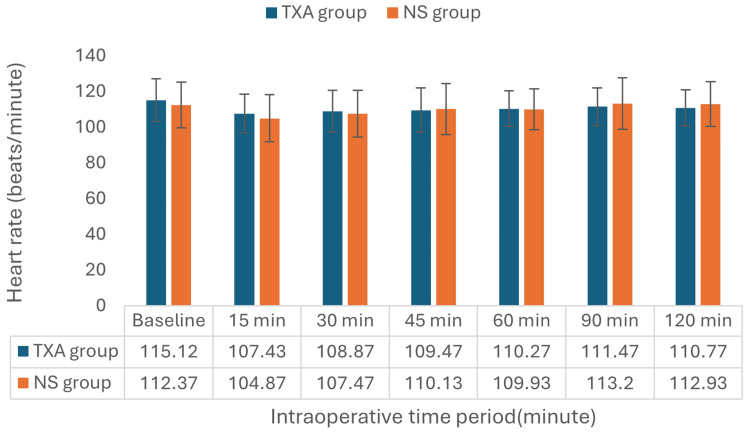
Intraoperative heart rate trend in both groups Values are expressed as mean. Baseline: Recorded just before being taken to the operation theater. TXA: tranexamic acid; NS: normal saline.

The intraoperative heart rates of the TXA and NS groups at various points throughout the procedure are displayed in Figure 2. The baseline heart rates were 115.10±11.97 beats per minute (bpm) for the TXA group and 112.37±12.72 bpm for the NS group (p=0.395). These heart rates stayed similar throughout the intraoperative phase, as indicated by the measurements taken at 15, 30, 45, 60, 90, and 120 minutes.

**Figure 3:**
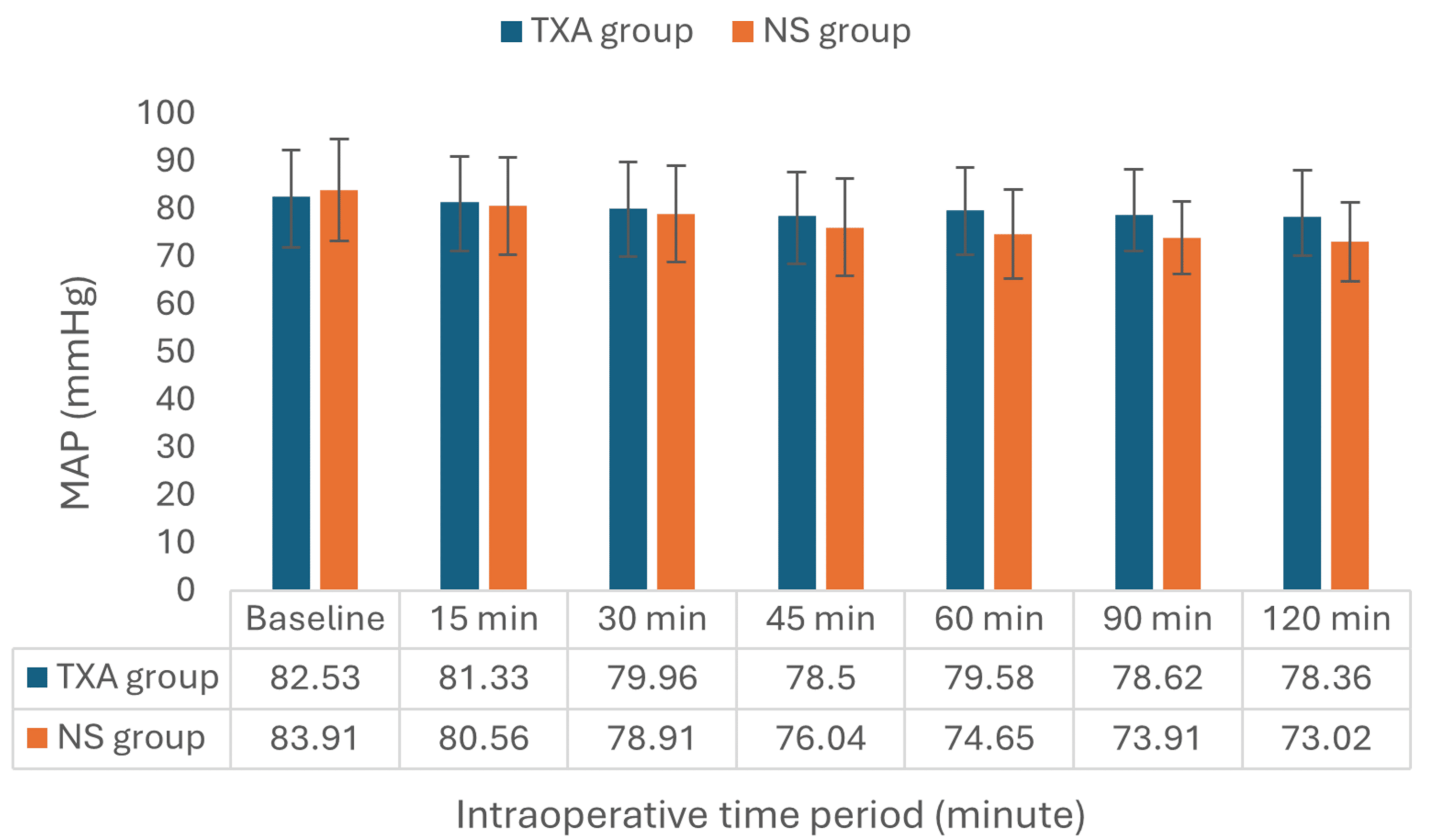
Intraoperative mean arterial pressure trend in both groups Values are shown as mean. Baseline: Recorded just before being taken to the operation theater. TXA: tranexamic acid; NS: normal saline; MAP: mean arterial blood pressure.

The TXA and NS groups' intraoperative MAP at different points during surgery are compared in Figure 3. At baseline, the TXA group's MAP readings were 82.53±9.76 mmHg, while the NS group's readings were 83.91±10.64 mmHg (p=0.603). Throughout the intraoperative phase, no statistically significant variation was observed in the MAP.

## Discussion

The present study enrolled 60 children, evenly randomized into an interventional (TXA group) and a placebo control (NS group). Our literature search indicates that this is the first randomized controlled trial evaluating the effect of TXA on bleeding during Wilms tumor surgery, a common surgery for neoplasm in children.

Both groups were comparable with respect to baseline characteristics like age, height, and weight, BMI, gender, ASA physical status, platelet count, INR, and serum creatinine (p > 0.05). These findings indicate group comparability and a minimum effect of confounding factors. Group comparability in parameters like BMI and weight is desirable to ensure that the difference in the amount of total blood loss can be attributed to the intervention.

In our study, the mean duration of surgery in the TXA group was 132.0 ± 19.4 minutes, compared to 138.0 ± 13.75 minutes in the saline group. This small difference in the duration of surgery was not statistically significant. Previous research also shows that prophylactic TXA in the pediatric population doesn’t create a significant difference in the time span of surgery [17-21].

We found that the average blood loss during surgery for the group that received TXA was lower than that in the control group (216.3 mL vs 306.4 mL), and this difference is statistically significant (p < 0.001). This finding underlines the effectiveness of TXA in decreasing blood loss during Wilms tumor surgery in children. These results align with the previous studies demonstrating TXA's efficacy in reducing blood loss during cranial, scoliosis, and craniosynostosis surgery [16-21]. In contrast, some studies on tonsillectomy and nasal sinus surgery cases have shown no significant improvement regarding blood loss, indicating that TXA may be less effective in airway surgeries [21,22].

Results from our study also indicate a significant reduction in the transfusion requirements in patients who received prophylactic TXA (167.1±24.9 mL vs 199.8±23.3 mL), showing its effectiveness in perioperative blood loss reduction during Wilms tumor surgery. These results are supported by Fenger-Eriksen et al. (2019), who found that patients receiving TXA needed 8.2 mL/kg of PRBC transfusion compared to 14 mL/kg (p = 0.01). ROTEM results indicated better maintenance of maximum clot firmness [17]. Reductions in transfusion amounts were also found in the studies by Chauhan et al. [23] and Bulutcu et al. [24].

In our study, urine output was higher in the TXA group, probably reflecting better intravascular preservation and renal perfusion. Serum lactate levels were significantly lower in the TXA group (1.82 mmol/L compared to 2.63 mmol/L), indicating better hemodynamic stability, tissue perfusion, and oxygen supply. Kim et al. (2021) also studied the role of TXA in tibial osteotomy surgery cases and found better urine output and less surgical drain blood loss in the TXA group [25]. 

In our study, the intraoperative heart rate and MAP values were comparable in both groups. Also, no thromboembolic or adverse events were observed in either group. This shows that TXA is safe for children, which matches what Fenger Eriksen et al. found in their study [17].

Limitations

This single-center design may have selection bias and reduce external validity. Not measuring TXA serum levels prevented us from understanding the potential correlation between varying plasma levels and the associated clinical effect. Another limitation is that not all surgeries were performed by a single surgeon, so surgical skill and technique could have affected the amount of blood loss. Additionally, a short follow-up period may miss delayed complications. Considering these, we recommend further multicenter trials based on this population.

## Conclusions

The result from this study indicates that prophylactic intravenous TXA during open nephrectomy helps in reducing intraoperative surgical blood loss without associated adverse events. Patients who received TXA also needed fewer amounts of PRBC transfusions and displayed less increase in serum lactate levels due to a reduction in blood loss.

So, we recommend the use of intravenous TXA as a part of surgical bleeding prophylaxis management in patients undergoing nephrectomy for Wilms tumor.
